# Using the Digital Flow to Increase Efficiency in Complex Partial Rehabilitation with Dental Implants

**DOI:** 10.1155/2022/7525837

**Published:** 2022-02-09

**Authors:** Andrew Sotirios Melenikiotis, Camila Pereira Vianna, Waleska Caldas, Larissa Carvalho Trojan, Rubens Moreno de Freitas

**Affiliations:** ^1^Implantology at Ilapeo College, Curitiba, Brazil; ^2^Pathology, Curitiba, Brazil; ^3^Orthodontics, Curitiba, Brazil; ^4^Biomechanics, Curitiba, Brazil; ^5^Ilapeo College, Curitiba, Brazil

## Abstract

**Background:**

This report presents a clinical case in which the CAD-CAM technology was applied to optimize a complex partial rehabilitation with implant-supported prostheses of a patient with several functional and aesthetic issues. *Case presentation*. A 40-year-old patient with several complaints due to the absence of multiple teeth and great dissatisfaction regarding his oral health was referred to a Dental College (Curitiba, Brazil). Guided surgery of 11 implants was planned. Digital flow and immediate loading protocol were applied. The patient was followed up for 2 years presenting good clinical and radiographic outcomes.

**Conclusions:**

The digital flow brought agility and precision to implant placement, immediate provisionalization added to satisfaction in the provisional phase, and CAD/CAM technology provided predictability and comfort to deliver the definite restorations.

## 1. Introduction

Computer-aided design and manufacturing (CAD-CAM) was developed in the 1960s and first applied in automotive industries. Later, it started to be used in dentistry, increasing digital flow popularity in dental offices every year, contributing to the preliminary stages of dental implant treatment such as diagnosis and planning, and the actual surgical and prosthetics procedures [1]. Among the advantages of its use are reduced time and production costs, high-quality restorations due to consistent precision, and reproducible results [2].

The integration of this technology with rapid prototyping methods made surgical guide fabrication possible, and its use for dental implant placement is referred to as guided implant surgery [3]. Guided surgeries allow predictability in the relationship between planned restorations and the underlying bony anatomy, providing the placement of multiple dental implants, according to surrounding anatomy and principles of ideal implant positioning and spacing. Thereby, this technology is especially beneficial in situations of multiple units or full arch immediate rehabilitation, with or without extractions [4].

The aim of this report is to present a clinical case in which the CAD-CAM technology was applied to optimize a complex partial rehabilitation with implant-supported prostheses of a patient with the absence of multiple teeth and several functional and aesthetic issues.

## 2. Case Presentation

**Figure 1:**
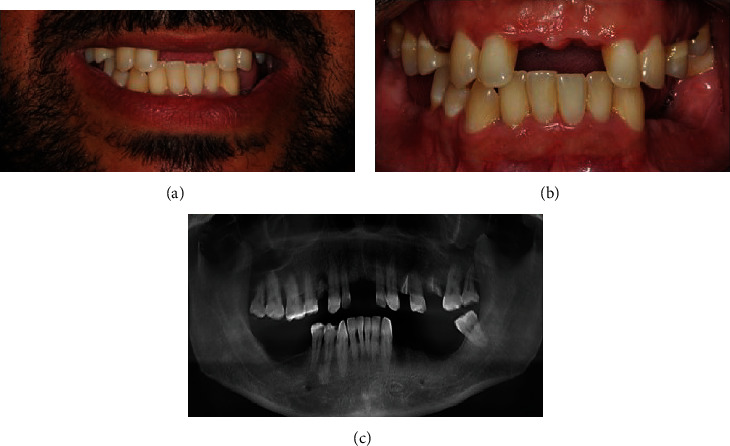
Patient's initial photos (a, b) and panoramic radiography (c) showing the absence of several elements in both arches.

A 40-year-old patient in good general health was referred to a Dental College (Curitiba, Brazil) with chief complaints of difficulty in eating/chewing due to the absence of multiple teeth, altered speech due to use of a removable provisional partial prosthesis with palatal extension, and low self-esteem due to dissatisfaction with smile's aesthetics and bad breath. After careful anamnesis and clinical and radiographic evaluation, it was also possible to diagnose the loss of teeth 11 due to caries and failed endodontic and restorative treatments (Figure 1). Verbal explanations of treatment options like a removable partial denture, conventional nonguided implant surgery, and rehabilitation with dental implants using the guided surgery technique were given, and the latter was the patient's choice due to enhanced comfort and reduced surgery time and morbidity. The patient gave written consent, and ethics approval was not necessary for this study.

### 2.1. Case Planning

**Figure 2:**
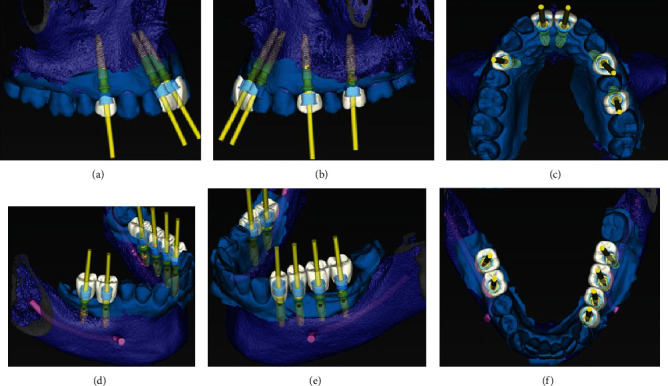
Virtual planning of maxillary (a)–(c) and mandibular (d)–(f) implants position considering the planned prosthesis rehabilitation.

Superior and inferior impressions were taken and later digitized with a lab TRIOS scanner and the STL files, together with the DICOM files of a full mouth CBCT, and were uploaded to Neodent website for guided surgery planning. These were processed in Dental Wings program of coDiagnostix software (Chemnitz, Germany) by a third party, who virtually planned the case according to instructions given by the oral surgeon (Figure 2). Once this plan was reviewed and approved by the oral surgeon, the surgical guide was fabricated with a 3D printer (Rapid Shape GmbH, Heimsheim, Germany).

### 2.2. Surgical and Prosthetic Procedures

**Figure 3:**
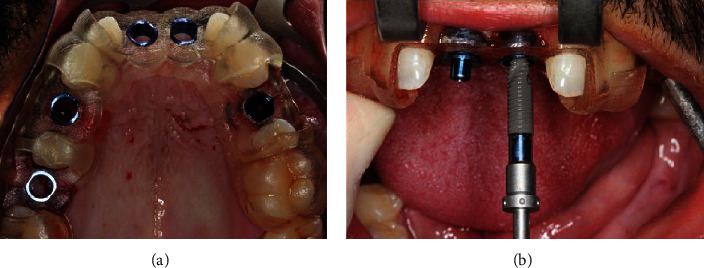
Tooth-supported surgery guide positioned in maxilla (a). Implant driver with stopper that indicates when the implant reaches the planned position (b).

The patient was medicated and prepared for surgery, and after local anesthesia, the residual roots of teeth 14, 24, and 26 were carefully extracted with minimally invasive techniques, after which the tooth-supported surgical guide was installed (Figure 3(a)), verifying a perfect fitting through the inspection windows. To increase the stability of the guide, stabilizing pins were screwed through the sleeves into the implant connection with the specific Guided Surgery Surgical kit for Helix GMimplants (Neodent, Curitiba, Brazil), and each bed was prepared following the sequence recommended by the manufacturer for bone type III, with the compensated drills through the corresponding drill guide and sleeve, whereas the drills have laser markings, the depth is controlled visually, and the implant drivers are fabricated with stoppers (Figure 3(b)). Once those touch the sleeve, they indicate that the implant has reached its planned position, which for this case was 2 mm subcrestal for all implants. Hydrophilic Morse cone tapered with 3.75 mm of diameter implants (Helix GMAcquaimplants, Neodent) and lengths between 8 and 13 mm were inserted in the sequence #14, 24,11,12,and 26. Once concluded, the stabilizing pins were removed as was the surgical guide, showing very little bleeding. The torque was measured with the torque wrench, and since all presented torques above 32 N/cm, indicating ideal primary stability for immediate loading, definitive screw-retained abutments were selected for each position, by means of measuring the transgingival height, with the aid of the GM height measurer and the abutment selection kit, and installed according to the specifications and torques recommended by the manufacturer (Neodent, Curitiba, Brazil). After this, and as previously planned, a technique for connective tissue graft in # 14 and xenograft fill was performed. Following the same steps as with the upper arch, implants in positions #35, 36, 37, 46, and 47 were placed using the surgical guide.

**Figure 4:**
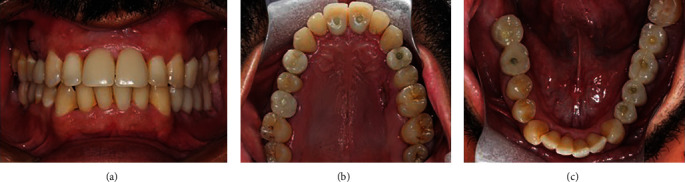
Clinical aspects at the time of immediate loading. Frontal view in occlusion (a), maxillary (b), and mandibular (c) occlusal view of acrylic resin temporary crowns.

**Figure 5:**
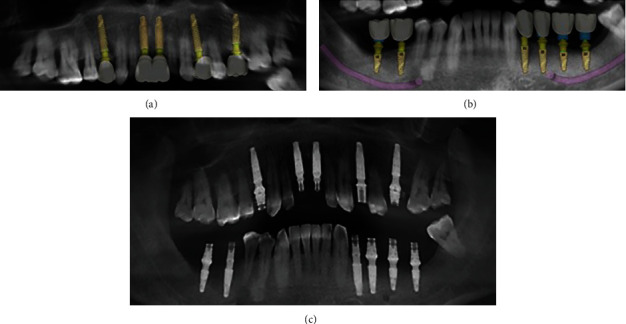
Virtual planning of implants and abutments (a, b) and immediate loading panoramic radiography (c).

In order to follow the one abutment-one time philosophy [5] and since a compatible digital impression coping (scan body) was not available at the time, the abutment impression copings were inserted for a conventional impression of both arches with addition silicone material (Kulzer, Hanau, Germany). Then, with the bite registration, it was sent to the lab for fabrication of the temporary crowns and bridges. The patient returned the following day to install the temporary acrylic restorations. The maxillary prostheses were single crowns, either screw-retained or with the click abutment for temps, and the mandibular ones were screw-retained multiunit acrylic prostheses (Figures 4 and 5).

**Figure 6:**
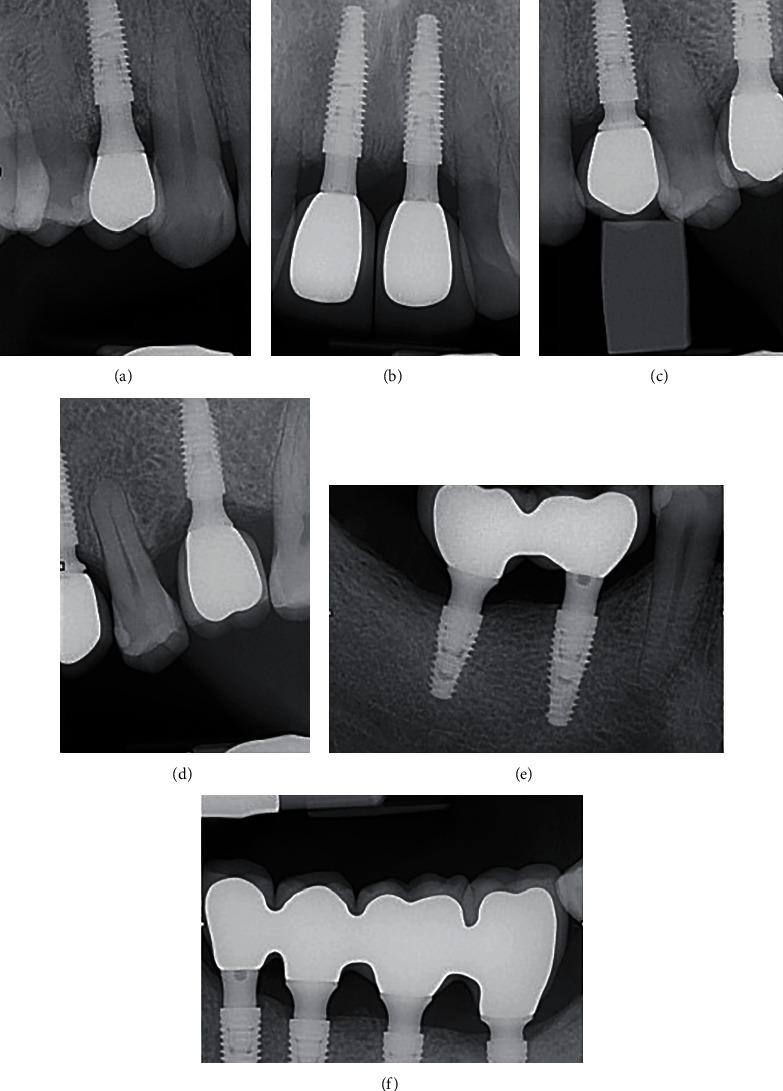
Periapical X-rays after maxillary (a)–(d) and mandibular (e, f) definitive prosthesis installation.

Three months thereafter, clinical and radiographic evaluation of the implants, abutments, and temporary prosthesis revealed adequate regeneration of peri-implant bone and soft tissues and, thus, the procedures for the confection of the final metal-ceramic restorations were followed through. Since the scan body for the GM conical abutment was not yet available, the GM conical abutments were replaced for GM Titanium Bases in positions # 14 and 26, to allow obtaining digital impressions. The scan bodies were inserted, and an intraoral scan was performed. Next, the metal coping try-in was conducted with radiographic verification, registration with red pattern resin (GC America, Alsip, USA), and repositioning of the temporaries. The metal copings were correctly positioned on the hybrid analogs in the printed models, with the resin registration and sent to the lab for porcelain build-up. After crownstry-in to adjust contact points and occlusal contacts, they were cemented onto the Tibases and then were installed (Figure 6).

The Portuguese translation of OHIP–14 (Oral Health Impact Profile) questionnaire [6, 7] was used to evaluate the quality of life related to oral health, as a measure of patient satisfaction during the treatment. The patient was asked before treatment, and after 6, 12, and 24 months postsurgery, how often, in the prior 6 months, he presented the problems evaluated by the questionnaire.

### 2.3. Clinical Follow-Up and Outcomes

The advantage of conducting the presented treatment using the guided surgery technique was mainly the ideal positioning of the implants, according to what was virtually planned, through evaluation of the virtual wax-up and available bone volume. Second, the ease and speed for site preparation and insertion of multiple implants, which, in conventional open-flap surgery, would have probably taken up to 4 sessions. Also, by having the virtual planning on the computer, communication with the patient by means of understanding the treatment plan was optimized, and the fascination factor by an innovative technology was decisive for this patient. During the surgery, the flapless technique resulted in little bleeding, the patient reported no postsurgical pain, and no edema was evidenced the days after, as well as other adverse events.

Regarding patient's satisfaction, at the screening visit, the subject reported to have had fairly often trouble pronouncing words and discomfort to eat. Besides that, life was considered less satisfied by him. Self-consciousness, tenseness, unsatisfactory diet, and embarrassment were reported by him to happen very often due to his teeth problems. Occasionally, the patient has had painful aching in his mouth, according to the questionnaire. Therefore, OHIP-14 score was 14.9, showing how unsatisfied the patient was with his oral health. However, after 6 months of treatment start, OHIP-14 decreased to 0, revealing great satisfaction, which remained at the 2 years follow-up visit.

**Figure 7:**
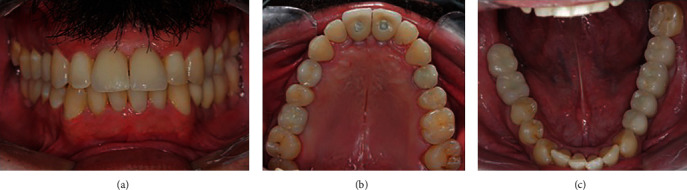
Clinical aspects at 2 years follow-up visit. Frontal view in occlusion (a), maxillary (b), and mandibular (c) occlusal photos.

**Figure 8:**
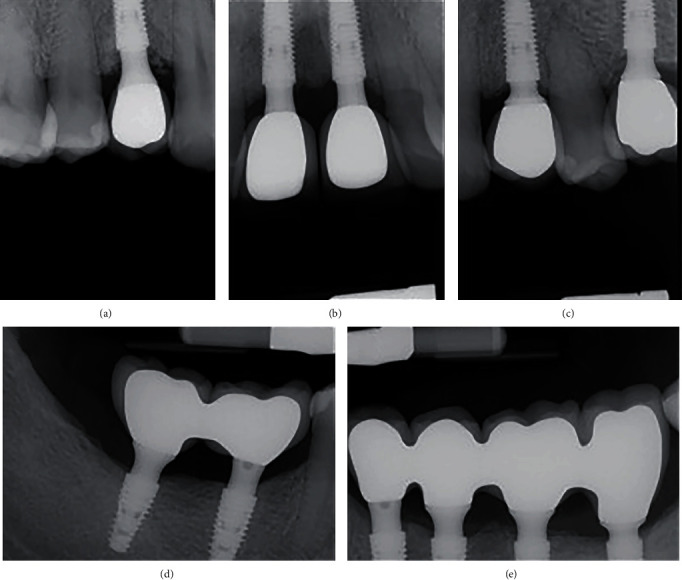
Periapical X-rays of maxillary (a)–(c) and mandibular (e, e) implants at the 24 months follow-up visit.

Also, good aesthetic outcomes remained 24 months after surgery, and soft tissue was clinically healthy (Figure 7). Moreover, no significant bone loss was observed at periapical X-rays, and thereby, all implants were considered as successful (Figure 8).

## 3. Discussion

The use of virtual planning and digital workflow has shown to improve preoperative planning and patients' comprehension of the proposed procedures, in addition to increased predictability and reduced surgical morbidity [3]. Moreover, the CAD/CAM technology makes a possible complex rehabilitation, like the one presented, to be completed with fewer appointments by anticipating surgical challenges and providing high quality restorations with consistent precision [1]. Also, regarding digital flow time-efficiency, it has been demonstrated that the entire clinical and laboratory process for single-unit crown production can take 16% less time than conventional prosthetic flow [8] Fewer clinical adjustments in digitally produced crowns have also been reported [2] and since these usually demand some time, digital flow can be very helpful, especially in complex cases with several prostheses to be installed.

Even though the present case was a guided surgery, surgeon's experience with conventional open flap free-hand surgeries, as well as passing through a good learning curve in the guided technique, was fundamental for the adequate management of the treatment. The understanding of the various steps and that errors in each step can accumulate and lead to the lack of precision of the final implant positioning, compared to the virtual planning, needs to be considered. First, careful image acquisition and reconstruction of CBCT and scanning, as well as segmentation and superpositioning of these images in the planning software, are performed. Then, adequate virtual wax-up, positioning of implants in relation to the wax-up, and choosing sleeves, followed by the design of the surgical guide, were also performed. After that, the virtual planning should be meticulously reviewed by the surgeon for approval. It is also important to assure careful maintenance of the 3D printer and correct printing and insertion of sleeves in the surgical guide, especially for these not to get loosen during the surgical procedure. Finally, previous disinfection/sterilization of the surgical guide without causing deformation should be made ,and the perfect fit of the tooth supported surgical guide should be verified through the inspection windows.

In comparison to conventional open-flap free-hand surgery, other advantages include during surgery, less bleeding, greater precision, and absence of suture; in the postoperative, less edema and pain, and consequently fewer medications leading to greater patient comfort and satisfaction; and in the healing process, since the periosteum is displaced, maintaining the blood supply, there is less bone and soft tissue, leading to an excellent prognosis and predictability [9], whereas some authors have shown that flapless surgery resulted in less crestal bone loss than when flaps are elevated [10]; other studies have reported that there was no statistically difference in bone loss between different techniques [11].

Flapless surgery has some drawbacks reported in the literature as the real condition of the underlying bone cannot be observed due to gingival tissue not being raised, which could lead to unwanted perforations and fenestration [12]. Another disadvantage that has been discussed is the potential for thermal damage due to reduced access for external irrigation [10]. However, even in the absence of flap, limited vision, and access to the bone bed, none of the aforementioned events occurred in the present case.

Finally, at the screening visit, the patient's OHIP-14 score was very high, revealing that his oral problems had a significant impact on function and social well-being, as reported in the literature [13]. However, after surgery, and being maintained for the 2 years of follow-up, the patient's great satisfaction showed how the implant-supported rehabilitation improved his quality of life.

## 4. Conclusion

With the use of technology and the digital flow, the prosthetically driven virtual planning for the correct 3D positioning of the implants provided the surgeon with a clear understanding of the outcome of this complex case with multiple implants in both arches, whereas the images generated an efficient communication with the patient. The guided surgery technique for implant placement, combined with the immediate loading concept with immediate provisionalization, brought agility and great comfort to both the patient and the professional. The intra-oral scanning for digital impression and CAD/CAM technology for design and fabrication of the definite restorations contributed to greater efficiency in re-establishing function and aesthetics to this oral rehabilitation case. A learning curve is necessary to understand all the steps involved in the digital flow.

## Data Availability

The data used to support this study are included within the article.
